# Nonsynonymous, synonymous and nonsense mutations in human cancer-related genes undergo stronger purifying selections than expectation

**DOI:** 10.1186/s12885-019-5572-x

**Published:** 2019-04-16

**Authors:** Duan Chu, Lai Wei

**Affiliations:** College of Life Sciences, Beijing Normal University, No. 19 Xinjiekouwai Street, Haidian District, Beijing, China

**Keywords:** Mutation, Cancer-related genes, Coding region, Codon usage bias (CUB), Natural selection

## Abstract

**Background:**

Nonsynonymous mutations change the protein sequences and are frequently subjected to natural selection. The same goes for nonsense mutations that introduce pre-mature stop codons into CDSs (coding sequences). Synonymous mutations, however, are intuitively thought to be functionally silent and evolutionarily neutral. Now researchers know that the optimized synonymous codon usage is advantageous in the speedy mRNA translation process. With the advent of NGS technique, the explosion of NGS data generated from the tumor tissues help researchers identify driver mutations in cancer-related genes, but relatively less attention is paid to the SNP data in healthy human populations when studying cancer.

**Methods:**

Here, we analyzed the publically available human SNPs. We classified these SNPs according to their functional and evolutionary categories. By simply dividing the human genes into cancer-related genes and other genes, we compared the features of nonsynonymous, synonymous and nonsense mutations in these two gene sets from multiple aspects.

**Results:**

We provided lines of evidence that the nonsynonymous, synonymous and nonsense mutations in cancer-related genes undergo stronger purifying selection when compared to the expected pattern in other genes. The lower nonsynonymous to synonymous ratio observed in cancer-related genes suggests the suppression of amino acid substitutions in these genes. The synonymous SNPs, after excluding those in splicing regions, exhibit preferred changes in codon usage and higher codon frequencies in cancer-related genes compared to other genes, indicating the constraint exerted on these mutations. Nonsense mutations are less frequent and located closer to stop codons in cancer-related genes than in other genes, which putatively minimize their deleterious effects.

**Conclusion:**

Our study demonstrated the evolutionary constraint on mutations in CDS of cancer-related genes without the requirement of data from cancer tissues or patients. Our work provides novel perspectives on interpreting the constraint on mutations in cancer-related genes. We reveal extra constraint on synonymous mutations in cancer-related genes which is related to codon usage bias and is in addition to the splicing effect.

**Electronic supplementary material:**

The online version of this article (10.1186/s12885-019-5572-x) contains supplementary material, which is available to authorized users.

## Background

Natural selection exists among all living organisms [1]. Mutation is the major source of selection and adaptation [2–5]. In coding region of DNA, it is imaginable that the nonsynonymous mutations that change the protein sequences would undergo strong selection, and those nonsense mutations that cause pre-mature termination of protein synthesis are also highly deleterious and subjective to purifying selection. However, the selection on synonymous mutations for a long time was thought to be negligible or very weak. It was previously reported that synonymous mutations might play a role in driving human cancers [6]. The study has fully discussed the effect of synonymous mutations on splicing events, and proposed some awesome ideas like the mutations silent to the protein sequence are not always silent to the function [6]. It is perfectly fine to consider that the synonymous mutations (or nonsynonymous mutations as well) that changed the splicing pattern of cancer-related genes were consequently driving cancer biogenesis and subjected to natural selection [7–9]. However, while it is well known that the selection on nonsynonymous mutations is about the change of amino acids, one major force that acts on synonymous mutation is the codon usage bias (CUB) [10–21]. The impact of synonymous mutations is more profound than people used to think. Studies have found that CUB and synonymous mutations participate in human diseases like autism [22] and hemophilia B [23]. At the molecular level, the codon usage would affect many aspects in mRNA translation, like tRNA selection, decoding efficiency and the elongation rate [10, 14, 16, 17], which frequently acts as rate limiting step in translation elongation. Thus, it is comparably important to study the role of synonymous mutations that affect the codon usage bias in cancer-related genes. Together with the evidence of splicing changes, the profile of the consequences caused by synonymous mutations in cancers would be clarified.

Intriguingly, in the field of CUB, studies have shown that an obvious advantage of optimized codon usage is that it facilitates the cellular translation elongation process and is especially beneficial upon rapid cell growth [24, 25]. This interpretation reminds us of rapid tumor growth. If the cancer-related genes have advantages in codon optimality, there could be a possible mechanism that the cancer-related genes take this advantage to achieve speedy translation, and eventually lead to rapid cell growth. Briefly speaking, the consequence of nonsynonymous changes is qualitative while the effect of synonymous changes is quantitative to the final protein product.

The advent of advanced sequencing techniques helps researchers more accurately profile the different Omics of cancers [26, 27]. In the field of cancer research, many attentions have been payed to the detection of mutations in tumor organs/tissues/cells since this is the direct way to search for driver mutations that cause the cancer [28–31], and little is done in analyzing the SNP or population genomic data from normal/healthy individuals. Intriguingly, in the history of evolution, many non-adaptive species went extinct, and only those adaptive ones are still extant. But evolutionary biologist could always find evidence of fitness and adaptation from the genomic data of extant species rather than extinct species [32]. For cancer researches, analyzing the patient data is undoubtedly the most efficient and direct approach, but the accessibility of some newly emerged cancer data is a problem to the vast majority of scientific researchers. Thus, digging out some valuable information in cancer-related genes from the commonly available human genome data (or even data in other species) is suitable for most researchers or evolutionary biologists [2].

In this study, we retrieved the recent version of human SNP data from NCBI dbSNP (http://www.ncbi.nlm.nih.gov/SNP/) and downloaded the list of human cancer-related genes from cancer gene census website (CGC, https://cancer.sanger.ac.uk/census/). By analyzing these nonsynonymous, synonymous and nonsense SNPs in cancer-related genes versus other genes (supposed to be unrelated to cancer), we found strong evidence for purifying selection on mutations in cancer-related genes from many aspects. The nonsynonymous to synonymous ratio is significantly lower in cancer-related genes compared to that in other genes, suggesting that the nonsynonymous substitutions in cancer-related genes are suppressed. For synonymous SNPs, the codons after mutation in cancer-related genes are preferred and more frequently used in the genome compared to those in other genes. We also observed that the nonsynonymous or synonymous SNP sites in cancer-related genes are more conserved at DNA level. Interestingly, the nonsense mutations are less frequent and meanwhile located closer to the stop codons in cancer-related genes versus other genes, which probably reduced their deleterious effects. Our study revealed signals of purifying selection on nonsynonymous, synonymous and nonsense mutations in human cancer-related genes from the publically available database rather than some precious data from cancer patients. Our work should be attractive to common researchers especially the cancer experts and evolutionary biologists.

## Methods

### Data collection

We collected the recent version of all human SNP data from NCBI dbSNP (http://www.ncbi.nlm.nih.gov/SNP/) (Version: Build 150. Last downloaded: January 2018). The common and all SNPs are labeled distinctly in the website. The list of 719 human cancer-related genes (Additional file 1: Table S1) was downloaded from the latest version of cancer gene census website (CGC, https://cancer.sanger.ac.uk/census/). The reference genome sequences of human (*H. sapiens*, version hg19) and mouse (*M. musculus*, version mm10) were downloaded from UCSC Genome Browser (genome.ucsc.edu), and the reference genome of rhesus macaque (*M. mulatta*, Ensembl version v89) was downloaded from Ensembl Genome Browser (www.ensembl.org).

### Annotation of human SNPs

We annotated the SNP sites using the hg19 human genome downloaded from UCSC Genome Browser (genome.ucsc.edu). If a SNP hits multiple isoforms of the same gene, the transcript with the longest CDS (canonical transcript) was retained. The canonical transcript of each gene was defined by the software SnpEff (version 4.2) [33]. If a SNP does not hit any genes, it is annotated as intergenic. Genes with at least one common SNP in CDS are given in Additional file 2: Table S2. All the information of a given SNP in CDS including the position on CDS, the amino acid before and after mutation, were inferred from the output file of SnpEff.

### Conservation analysis

Conservation level of genomic positions is measured by phyloP score (file hg19.100way.phyloP100way.bw, downloaded from UCSC Genome Browser, genome.ucsc.edu). Briefly, sites with higher conservation level have higher phyloP scores. The orthologous sites (genomic coordinates) between human and mouse or between human and rhesus macaque were transferred with liftOver [34] based on the pairwise genomic alignments. The liftOver chain files were downloaded from UCSC Genome Browser website (http://hgdownload.soe.ucsc.edu/goldenPath/hg19/liftOver/). Bedtools (version 2.25) [35] was used to extract sequences of a give region according to the reference genome.

### Codon usage bias

The protocol of calculating codon usage bias was carried out by an early study [21]. The codon bias of a gene is calculated by the deviation (chi-square) of A/T content of synonymous codons from that of intronic regions. Higher deviation indicates stronger codon bias for a gene. Codon bias of a particular codon is the correlation coefficient between codon frequency within a synonymous codon family and the deviation (chi-square value) of each gene [21]. Higher correlation coefficient suggests stronger bias (preferred) for a codon.

We summarize the pipeline as follows:

1. The 59 sense codons (without ATG, TGG and three stop codons) are classified into 21 synonymous codon families. Leu (L), Arg (R) and Ser (S) have six codons so that a single mutation sometimes could not switch one codon to another (for example, AGT and TCT both encode Ser but a single mutation could not change one to another). Therefore, each of these amino acids has to be divided into two synonymous codon families [21].
2. For each of the synonymous codon family within each gene, we calculated the deviation (chi square) of A/T content at codon position 3 from the intronic A/T content (59% in human). Take Lys (K) codon AAG for instance, family “K” has two codons AAG and AAA. In gene X, let us suppose:
  O_AAA_ = observed number of AAA codons.
  O_AAG_ = observed number of AAG codons.
  E_AAA_ = (O_AAA_ + O_AAG_) × 0.59, the expected number of AAA codons (within family “K”).
  E_AAG_ = (O_AAA_ + O_AAG_) x (1–0.59), the expected number of AAG codons (within family “K”).
  Chi square (*X*^*2*^) _[family K, gene X]_ = chisq.test(matrix(c(O_AAA_, O_AAG_, E_AAA_, E_AAG_), ncol = 2))$ statistics. “chisq.test” is a function in R language (http://www.R-project.org/) for conducting chi square tests.
3. For each gene, the gene level CUB is the “scaled chi square”, which is the sum of the chi square values of the codon families within this gene normalized by peptide length. A higher “scaled chi square” value indicates stronger CUB for a gene.
4. For each of the 59 sense codons, the codon level CUB (codon preference) is measured by the Spearman’s *Rho*. Again, take Lys codon AAG for instance (the original literature also took this codon as an example, please refer to Akashi 1995 Fig. 1), family “K” has two codons AAG and AAA. The Y-axis is the frequency of AAG among “AAG + AAA” in each gene (each dot represents a gene). The X-axis is the corresponding scaled chi square value of each gene (Akashi 1995 Fig. 1). The codon level CUB (codon preference) is the Spearman’s *Rho* between the X-axis and Y-axis. *Rho* > 0 indicates a preferred codon and *Rho* < 0 indicates an unpreferred codon [21]. In human, the *Rho* value for codon AAG is 0.74.
5. This algorithm was implemented to all the human genes and all 59 sense codons. There is a tendency that G/C ending codons are preferred and A/T ending codons are unpreferred.

**Fig. 1:**
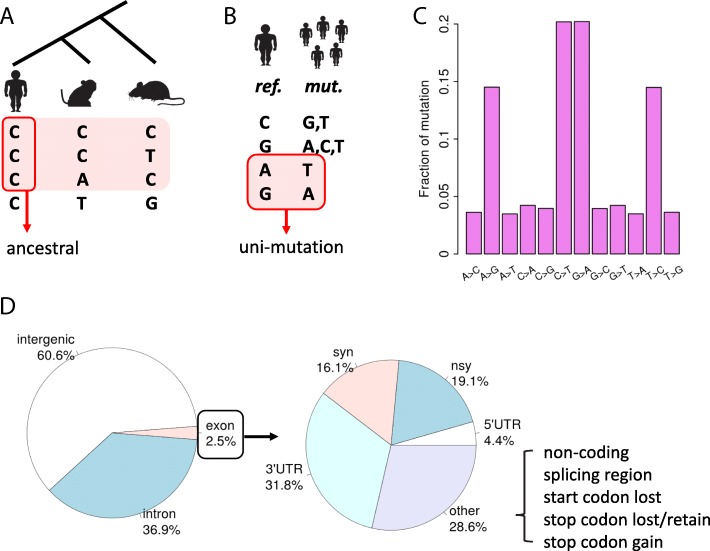
The landscape of human SNPs. **a** A schematic diagram showing the filtering of SNPs according to the orthologous sites in rhesus monkey and mouse genomes. The phylogenetic tree is unscaled. **b** A schematic diagram displaying the definition of uni-mutation SNPs. *ref*., reference. *Mut*., mutation. **c** the fractions of different types of mutations. **d** The annotations and proportions of genomic SNPs (left) and exonic SNPs (right) after the above filtering steps. The clip arts of human/monkey/mouse were drawn by ourselves

For a given codon, the delta codon bias (or similarly, delta codon frequency) is the difference between the codon bias values of “post-mutation” and “pre-mutation” versions. The delta codon bias (or similarly, delta codon frequency) of a gene is defined as the mean delta value of all the SNPs in the coding region of this gene.

### Calculation of gene expression level in human HeLa cells

We searched for the public database and chose an NGS dataset conducted in human HeLa cells (GES63591) [36]. The mRNA-Seq library (si-control) was used to define the gene expression level. We aligned the mRNA-Seq NGS reads to the hg19 reference genome using STAR (version 2.7) [37]. The uniquely mapped reads were kept for downstream analysis. The read counts of each gene were calculated by htseq-count (version 0.5.4) [38]. In this gene expression calculation, the canonical transcript of each gene was chosen, and all the reads overlapped with exon regions were counted. Highly expressed genes (9903 genes in total) are defined as gene with reads count > 100. Moreover, according to the SNPs we retrieved, there are 17,940 genes that have SNPs in their CDSs. 649 out of the 719 cancer-related genes (as aforementioned) belong to these 17,940 genes. When we only consider the 9903 highly expressed genes in HeLa cells, 8857 overlapped with the 17,940 genes with SNPs, and 439 of them belongs to cancer-related genes and 8418 are other genes. The analyses that take gene expression level into count are conducted using these 439 cancer-related genes versus 8418 other genes.

### Statistical analysis and code availability

All statistical analyses (correlation tests, Fisher’s exact tests, Wilcoxon rank sum tests) and the graphic work were conducted in R environment (http://www.R-project.org/) (version 3.3.3). All codes used in the analyses are available under request.

## Results

### The landscape of human SNPs

To get the full set of human SNP data, we retrieved the recent version of human SNPs from NCBI dbSNP (http://www.ncbi.nlm.nih.gov/SNP/). Among the “common” and “all” SNPs that are labeled distinctly in the website, we first chose the common SNPs for analysis due to its higher occurrence and reliability, since the total number of all SNPs already came up to one tenth of the human genome. In total, 34,082,224 common SNP sites were downloaded. We began to filter these common SNPs (Fig. 1a). We sought for the ancestral variants in human (*Homo sapiens*) genome according to the orthologous sites in two other mammalian species, rhesus macaque (monkey, *Macaca mulatta*) and mouse (*Mus musculus*). Only those SNPs with genomic sequences identical to at least one species in monkey or mouse were considered as ancestral SNPs (Fig. 1a). Next, part of the SNPs has more than one mutation types and might cause conflict in functional annotation. Thus, these “multi-mutation” SNPs were discarded and only those “uni-mutation” SNPs were retained (Fig. 1b). After filtration, 21,221,571 SNPs were remaining, among which the C > T and G > A transitions are the most prevalent while transversions are less frequent (Fig. 1c). We annotated the SNPs with SnpEff [33] and the canonical transcript of each gene were chosen. Most of the SNPs were located in intergenic or intronic region, and the exonic SNPs were dispersed in CDSs, UTRs or noncoding RNAs (Fig. 1d). There were totally 17,940 genes that has at least one SNP in CDS (Additional file 2: Table S2). Note that we have excluded those sites in splicing region when defining nonsynonymous or synonymous mutations in CDSs. Therefore, if any selection is detected for the nonsynonymous or synonymous sites, it might not be imposed by the selection on splicing events.

### Profiling the SNPs in cancer-related genes and other genes

We downloaded the list of human cancer-related genes (719 genes, Additional file 1: Table S1) from the latest version of cancer gene census website (CGC, https://cancer.sanger.ac.uk/census/), and 649 of them belong to the 17,940 genes and have SNPs in CDSs. In the following analyses, the genes with SNPs in coding regions were divided into cancer-related genes and other genes. First we calculated the SNP density (number of SNPs per Kb) in exonic (Additional file 3: Figure S1A) or coding (Additional file 3: Figure S1B) region. The result is that the SNPs in cancer-related genes are strongly avoided especially those in CDSs (Additional file 3: Figure S1B). Furthermore, we displayed the global profile of number of SNPs in each functional category, in cancer-related genes and other genes, respectively (Additional file 3: Figure S1C). It is reasonable that many SNPs in other genes belong to the “other” category, the majority of which are noncoding RNAs. Since the identified cancer-related genes are mainly protein-coding genes, the noncoding RNAs are consequently grouped to the other genes.

### Constraint on mutations belonging to different evolutionary categories

**Fig. 2:**
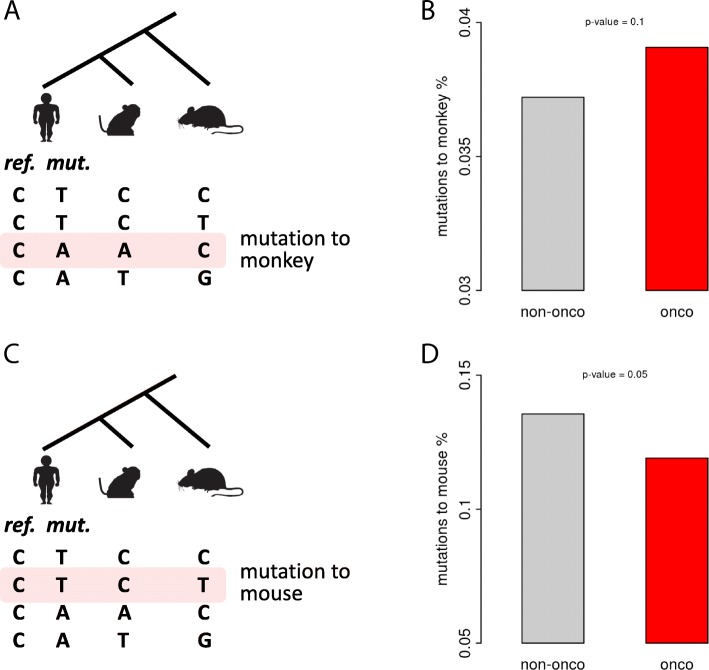
Constraint on mutations belonging to different evolutionary categories. **a** A schematic diagram showing the definition of mutation to monkey. The phylogenetic tree is unscaled. **b** Fractions of SNPs that the reference allele is observed in mouse and the alternative allele is observed in monkey. *P*-value was calculated using Fisher’s exact test. **c** A schematic diagram showing the definition of mutation to mouse. The phylogenetic tree is unscaled. **d** Fractions of SNPs that the reference allele is observed in monkey and the alternative allele is observed in mouse. *P*-value was calculated using Fisher’s exact test. “onco” denotes cancer-related genes; “non-onco” denotes other genes. The clip arts of human/monkey/mouse were drawn by ourselves

We have profiled the SNPs in different functional categories and found that the mutations in CDS of cancer-related genes are constrained. This might not be new to us since we did not separate nonsynonymous and synonymous sites. Before we go deep into the selection on nonsynonymous and synonymous SNPs, we tried to reveal the selection in CDS of cancer-related genes from another aspect. We defined “mutation to monkey” as the human SNPs with reference allele identical to the mouse genome and the alternative allele identical to the monkey genome (Fig. 2a). We found that the cancer-related genes have higher fraction of this kind of SNPs (Fig. 2). On the contrary, for the “mutation to mouse” as we definebd (Fig. 2c), they are significantly avoided in cancer-related genes (Fig. 2d). For those mutations to mouse, the reference allele in human should already be fixed in the common ancestor of human and monkey, so the novel mutation in human might be deleterious and depleted in cancer-related genes (Fig. 2c, d). But for the mutations to monkey, the reference allele in monkey should already be fixed in the common ancestor of human and monkey, and the human reference allele might be the deleterious one. Thus, the changes caused by human SNPs simply “reversed” the deleterious allele in human genome, which might be welcome by the cancer-related genes (Fig. 2a, b).

### Purifying selection on the nonsynonymous as well as the synonymous SNPs in cancer-related genes

**Fig. 3:**
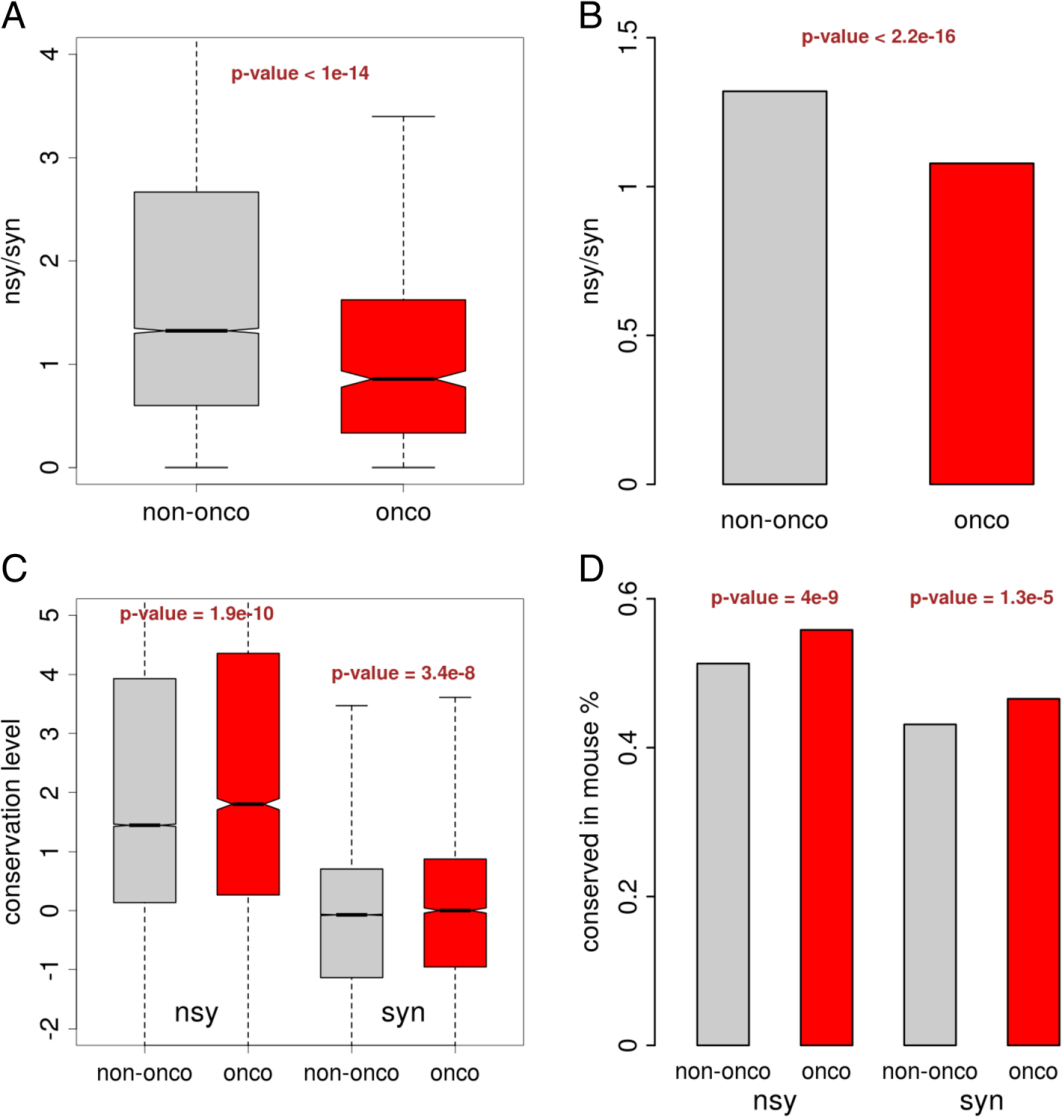
Purifying selection on the nonsynonymous as well as the synonymous SNPs in cancer-related genes. **a** Comparison between the nonsynonymous to synonymous ratio (nsy/syn) of cancer-related genes and other genes. *P*-value was calculated using Wilcoxon rank sum test. **b** Comparison between the pooled nsy/syn ratio of cancer-related genes and other genes. P-value was calculated using Fisher’s exact test. **c** Conservation level (phyloP score) of nonsynonymous and synonymous SNPs in cancer-related genes and other genes. *P*-values were calculated using Wilcoxon rank sum tests. **d** Fractions of conserved orthologous sites in mouse genome. *P*-values were calculated using Fisher’s exact tests. “onco” denotes cancer-related genes; “non-onco” denotes other genes

Nonsynonymous to synonymous ratio (nsy/syn) is a very commonly used measurement in evolutionary biology to detect the positive or purifying selection. From our SNPs data, we could see that the nsy/syn ratios in cancer-related genes are significantly lower than those in other genes (Fig. 3a for each gene and Fig. 3b for pooled result). This pattern suggests that nonsynonymous mutations in cancer-related genes are subjected to purifying selection and are repressed. Again, this might not be new to us since numerous driver mutations were reported to cause nonsynonymous changes. Next, we examined the conservation level of the nonsynonymous and synonymous SNP sites. Note that the conservation level of a site refers to the DNA level conservation pattern across different species (and it has nothing to do with the SNP). A very smart measurement is the phyloP score (downloaded from UCSC Genome Browser, genome.ucsc.edu) calculated from the multiple sequence alignment of a large set of species. We found that for both the nonsynonymous and synonymous sites in cancer-related genes have higher conservation level than those in other genes (Fig. 3c). Another aspect to reflect the DNA conservation level is to calculate the fraction of sites that are conserved between human and the orthologous sites in another species (e.g. mouse). Similarly, we observed higher conservation level for the nonsynonymous or synonymous sites in cancer-related genes compared to those in other genes (Fig. 3d). Taken together, the DNA sites of the nonsynonymous and synonymous SNPs are more conserved in cancer-related genes than other genes, indicating that the mutations on these sites might cost a higher price. This result supports our notion that both nonsynonymous and synonymous SNPs in cancer-related genes are subjected to purifying selection.

### Purifying selection on the synonymous SNPs in cancer-related genes

**Fig. 4:**
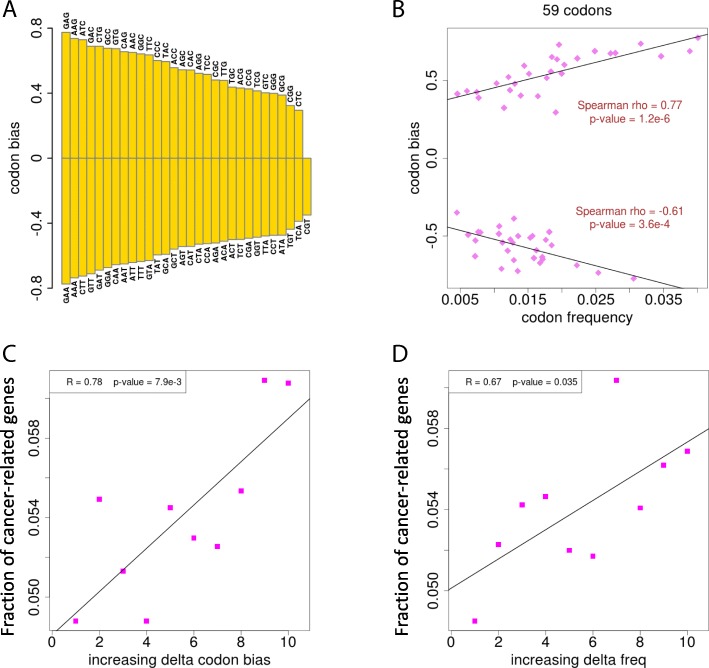
Purifying selection on the synonymous SNPs in cancer-related genes. **a** Codon bias (codon preference) of the 59 sense codons in human genome. Codons with a positive value are preferred, and vice versa. **b** Correlation between codon bias and codon frequency appearing in human genome. **c** Correlation between the delta codon bias after the synonymous change and the fraction of cancer-related genes. Genes were divided into ten bins with increasing delta codon bias. **d** Correlation between the delta codon frequency after the synonymous change and the fraction of cancer-related genes. Genes were divided into ten bins with increasing delta codon frequency. “onco” denotes cancer-related genes; “non-onco” denotes other genes

As we have mentioned above, the major selection force acting on synonymous mutations is the codon usage bias. We followed an early study [21] and calculated the parameters needed for codon bias (see Methods for detail). In brief, each of the 59 sense codons (excluding three stop codons and two codons without synonymous counterparts) has a “codon bias value” between − 1 and 1 (which is actually a correlation coefficient). A positive value indicates a preferred codon, and vice versa. The extent of preference increases with this codon bias value (Fig. 4a). As clearly shown, the preferred codons are C/G ending and those unpreferred ones are A/T ending. But do not simply treat this codon bias value as the frequency of each codon appearing in the genome. The codon bias and codon frequency is highly correlated in the preferred or unpreferred groups separately, but not together (Fig. 4b). Therefore, the codon bias and codon frequency could be used as independent parameters to measure a synonymous change. For each synonymous human SNP, we calculated the change (delta) of codon bias and codon frequency after mutation. We divided all these synonymous sites into ten bins with the order of increasing delta codon bias (Fig. 4c) or delta codon frequency (Fig. 4d). In each bin, we calculated the fraction of cancer-related genes. Amazingly, the fractions of cancer-related genes are significantly positively correlated with the bins of delta codon bias (Fig. 4c) or delta codon frequency (Fig. 4d). This trend demonstrates that the synonymous SNPs in cancer-related genes tend to gain a more frequently used codon or a preferred codon (compared to those in other genes). The result strongly suggests the purifying selection exerted on synonymous SNPs in cancer-related genes.

### Dilemma of cancer-related genes: lower nsy/syn ratios but preferred codon changes

We have demonstrated that the cancer-related genes have significantly lower nsy/syn ratios (Fig. 3), and that the synonymous or nonsynonymous mutations in cancer-related genes tend to increase the codon preference or codon frequency compared to other genes (Fig. 4). It is reasonably to ask that whether the nsy/syn ratio of genes and the changing direction of codon preference are correlated. Contrary to our expectation, at gene level, the nsy/syn ratio and the delta codon bias (Additional file 3: Figure S2A) or the delta codon frequency (Additional file 3: Figure S2B) is positively correlated. Here comes the dilemma of cancer-related genes. Cancer-related genes are the set of genes with low nsy/syn ratios but high delta codon bias/frequency values. We selected the cancer-related genes with relatively low nsy/syn ratios (nsy ≤ 2 and syn ≥ 2), and displayed the top 20 genes with the highest delta codon bias (Additional file 3: Figure S2C). These genes are supposed to suffer most from the dilemma. We also illustrated the relative position of the nonsynonymous or synonymous SNPs on the CDSs of these 20 genes (Additional file 3: Figure S2D). Given the dilemma of cancer-related genes, we could consider that the nsy/syn ratios and the codon usage might play independent roles in oncogenesis. This is not paradoxical because the nonsynonymous mutations contribute to cancer by changing the amino acid and the protein function, while synonymous mutations putatively contribute to cancer by affecting codon usage and the mRNA translation rate.

### Purifying selection on the nonsense mutations in cancer-related genes

**Fig. 5:**
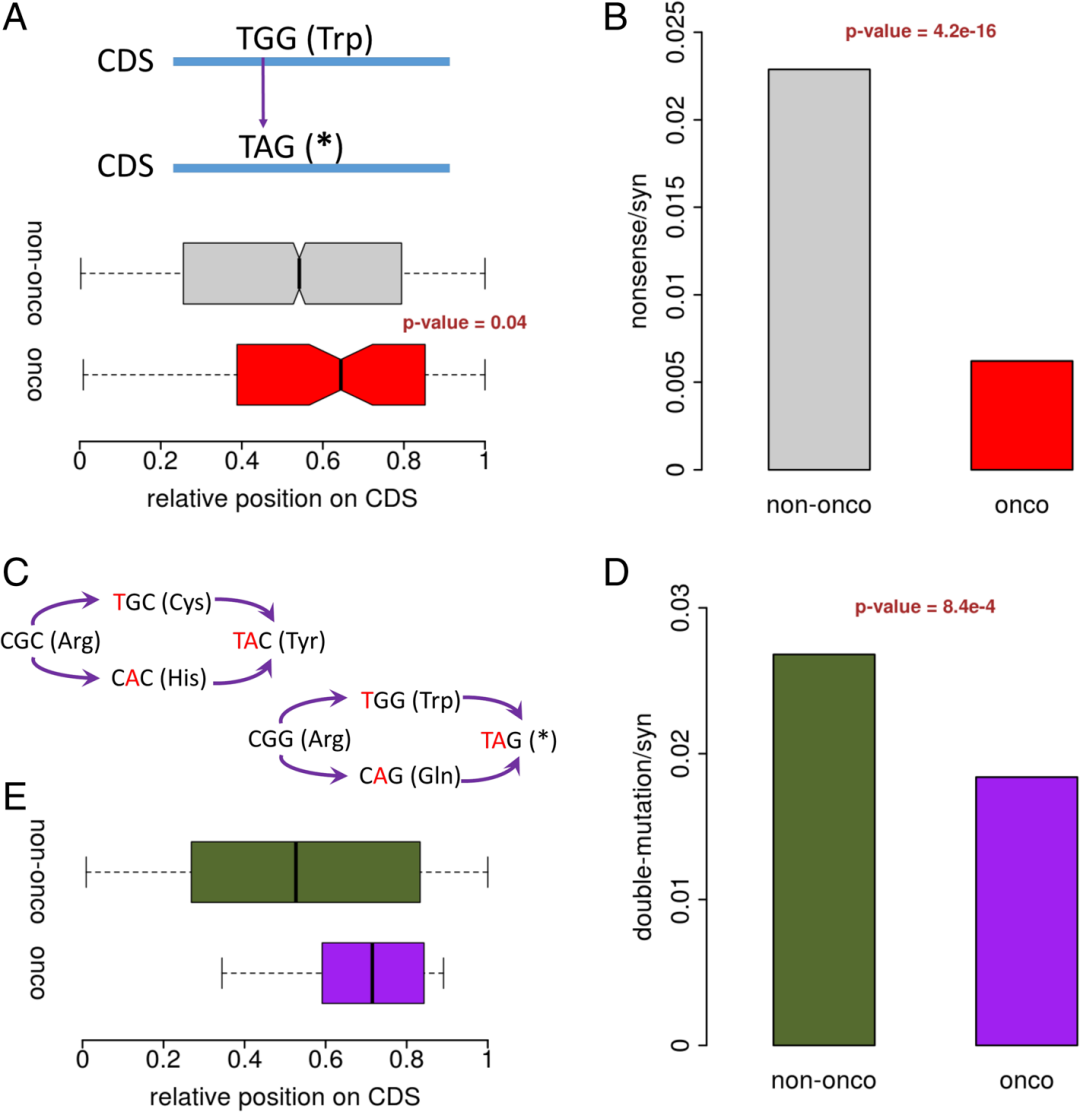
Purifying selection on the nonsense mutations that create stop codons in coding region. **a** Relative position of the nonsense variants in cancer-related genes and other genes. *P*-value was calculated using Wilcoxon rank sum test. **b** Comparison between the nonsense/syn ratios in cancer-related genes and other genes. P-value was calculated using Fisher’s exact test. **c** A diagram illustrating the effects of double-mutation within a codon. Quite a lot of double-mutations result in a stop codon in CDS. **d** Comparison between the double-mutation/syn ratios in cancer-related genes and other genes. *P*-value was calculated using Fisher’s exact test. **e** Relative position of the double-mutations that create stop codons cancer-related genes and other genes. “onco” denotes cancer-related genes; “non-onco” denotes other genes

Nonsense mutations introduce pre-mature stop codons to CDS, which are highly deleterious and subjected to purifying selection in most cases. Intriguingly, when we calculated the relative positions of nonsense mutations (SNPs) on CDS, we discovered that the nonsense mutations in cancer-related genes are located closer to the stop codons (Fig. 5a). If a “less truncated” protein is less deleterious, the nonsense mutations closer to the end of CDS would reduce their harms. Thus, our observation is probably the relics shaped by the purifying selection on the nonsense mutations in cancer-related genes. Moreover, similar to the nsy/syn ratio, we calculated the nonsense/syn ratios of cancer-related genes and other genes (Fig. 5b), unsurprisingly, the ratio in cancer-related genes is significantly lower. An interesting issue is that if we consider multiple mutations in the same codon simultaneously, the combined effect of the mutations might be different from any of the single mutations (Fig. 5c), and the combined effect can even lead to a nonsense mutation. Considering that the adjacent mutations are not necessarily coupled in the same individual, this concern of “multiple mutations” may not be useful in all cases. In this case of human SNPs, we extracted all the “double-mutations” that are located in the same codon, and selected the cases in which the combined effect is different from any of the single mutations (in total 691 cases). Amazingly, among all these 691 cases, 129 (18.7%) were causing nonsense mutations eventually. We calculated the double-mutation/syn ratios in cancer-related genes and other genes, and found that the double-mutations in cancer-related genes are significantly suppressed (Fig. 5d), probably due to their deleterious effect similar to that of nonsynonymous mutations. For the 129 cases of nonsense mutations caused by double-mutations, the cases in cancer-related genes are located closer to the end of CDSs (Fig. 5e). In this part, we fully demonstrated the purifying selection on nonsense mutations in cancer-related genes, and these so called nonsense mutations could even be derived from multiple nonsynonymous or synonymous mutations within the same codon.

### Other confounding factors to be addressed

Several confounding factors need to be considered before a consolidated conclusion is drawn. The factors are listed as follows: (1) While the cancer-related genes are well-annotated, the “other genes” might contain many poorly characterized genes which could introduce bias into the analysis; (2) GC content might potentially affect the results; (3) The canonical transcript chosen for each gene might not be representative or could be lowly expressed.

To address these problems, we first decided to take into account the gene expression level. We believe that the highly expressed genes (usually more conserved and more important) tend to be better annotated and those lowly expressed genes tend to be (relatively) poorly characterized. Therefore, if we only focus on the highly expressed genes and observe the same pattern (between cancer-related genes versus other genes), then the potential bias could be canceled.

We retrieved the highly expressed genes (NGS read count > 100, see Methods for details) in human HeLa cells. In total, 9903 genes are defined as highly expressed. We used this set of highly expressed genes and tested the following patterns between cancer-related genes versus other genes (Additional file 3: Figure S3): (1) Nonsynonymous to synonymous ratios; (2) Conservation level of SNP site; (3) The changes of CUB of synonymous mutations. The results are as follows: (1) The nonsynonymous to synonymous ratios in cancer-related genes are significantly lower than those in other genes; (2) Both nonsynonymous and synonymous mutations in cancer-related genes are more conserved at DNA level compared to mutations in other genes; (3) The synonymous mutations exhibit preferred changes in codon usage in cancer-related genes compared to other genes. These results indicate that even if we only look at the highly expressed genes (which are likely to be well-characterized), the pattern on nonsynonymous or synonymous mutations in cancer-related genes also exists.

To control for the effect of GC content, we chose the top 20% genes with the highest GC content (Additional file 3: Figure S4) and the bottom 20% genes with the lowest GC content (Additional file 3: Figure S5). Within each gene set, we compared the following issues in cancer-related genes versus other genes. (1) Nonsynonymous to synonymous ratios; (2) Conservation level of SNP site; (3) The changes of CUB of synonymous mutations. These patterns are the main points of our study. Fortunately, the patterns keep intact if we only focus on the high GC genes (Additional file 3: Figure S4) or low GC genes (Additional file 3: Figure S5), suggesting that the differences observed in cancer-related genes versus other genes are not caused by GC content of genes.

Next, we need to discuss the potential limitation if we only consider the canonical transcript of each gene. We think our control for gene expression level (Additional file 3: Figure S3) should partially cancel this bias because the gene expression level is calculated by NGS read count on the canonical transcript (Methods). Moreover, in some cases, different isoforms of the same gene share the same CDS, which might reduce the bias of this approach (since we only focus on the variations in CDS).

In addition, in the CUB analyses, one might concern that a comprehensive study should add analysis on (1) only the 4-fold degenerate sites and considering that (2) the mutations are context-dependent. Moreover, the patterns observed in nonsense mutations (between cancer-related genes versus other genes) might be related to CUB. These issues are untested at this stage and are regarded as limitations in this study.

## Discussion

Genomes of extant species are the relics shaped by natural selection and inform us the scenario after the selections happen. If the extinct species were resurrected, it would definitely help evolutionary biologists more accurately infer the positive and purifying selections. However, analyzing the available data is so far the most practical way to study evolution and infer natural selection. Similarly, investigating the genomic data of healthy individuals gives us another aspect to reflect the evolutionary constraint and selection events in cancers and cancer-related genes, even without the patient data from numerous cancer types. We could imagine that the constraint observed in the cancer-related genes of healthy individuals would indicate that the bad mutations in cancer-related genes might appear in some cancer types and not be observed by researchers.

Nonsynonymous mutations are thought to be largely deleterious due to their property of changing amino acids. The same goes for nonsense mutations that induce truncated proteins. The synonymous mutations that are originally thought neutral are now recognized to impact the codon usage bias and undergo natural selection as well. In this study, we retrieved the human SNP data and the list of human cancer-related genes. We removed the SNPs in splicing regions, which potentially excludes the effect of mutations on splicing patterns. We found very clear evidence for purifying selections on nonsynonymous, synonymous and nonsense mutations in cancer-related genes compared to the expected level inferred from other genes.

For nonsynonymous mutations, we observed that the nsy/syn ratio is remarkably lower in cancer-related genes compared to that in other genes. Among the synonymous SNPs, the codons after mutation in cancer-related genes tend to be preferred and have higher frequency in the genome compared to those in other genes. It is interesting that the optimized codon usage could facilitate the cellular translation elongation process and is beneficial upon rapid cell growth [24, 25]. It remains an open question that whether cancer-related genes took this advantage to achieve rapid tumor growth. The nonsense mutations in cancer-related genes are less frequent and meanwhile located closer to the end of CDSs. Interestingly, these nonsense mutations could be caused by either a single mutation or double (nonsynonymous) mutations within the same codon. At the whole CDS level, we discovered that in cancer-related genes, the mutations towards mouse are suppressed and the mutations towards monkey are favored. We also observed that the nonsynonymous or synonymous SNP sites in cancer-related genes are more conserved (at DNA level) and less polymorphic than those in other genes.

For the limitation of this study, we have aforementioned that the poorly annotated gene sets, the GC content and the choice of canonical transcripts might introduce biases to our results. However, we have tested our main points and patterns when these confounding factors are controlled. Thus, our results are consolidated at this stage.

Our study reported the signals of purifying selection on nonsynonymous, synonymous and nonsense SNPs in human cancer-related genes from multiple aspects. Although the deleterious effects of nonsynonymous and nonsense mutations are obvious, we indeed reflect these effects in different ways. Moreover, in addition to the known effect of synonymous mutations on mRNA splicing [6], we displayed the selection on codon usage bias for synonymous SNPs in cancer-related genes. Importantly, in cancer diagnosis, attentions have been paid to the detection of nonsynonymous (or nonsense) driver mutations. Here, with the evidence of purifying selection on synonymous SNPs in cancer-related genes, analyzing these sets of “silent” mutations should be equally crucial in predicting the risk of cancer, and some rare synonymous mutations in cancer-related genes might even be the direct targets in cancer therapy. Our work should be interesting to the cancer research community and the field of evolutionary biology.

## Conclusion

Our study demonstrated the evolutionary constraint on mutations in CDS of cancer-related genes. We drew to this conclusion without the requirement of data from cancer tissues or patients. We reveal extra constraint on synonymous mutations in cancer-related genes which is related to codon usage bias and is in addition to the splicing effect. The optimized codon usage in cancer-related genes might contribute to rapid cell growth and could be a potential mechanism related to oncogenesis.

## Additional files

Additional file 1: **Table S1.** List of 719 human cancer-related genes. (XLSX 18 kb)

Additional file 2: **Table S2.** Genes with at least one common SNP in CDS. (XLSX 220 kb)

Additional file 3: **Figure S1.** Profiling the SNPs in cancer-related genes and other genes. **a** Exonic SNP density in cancer-related genes and other genes. The density is calculated by the number of exonic SNPs divided by the length (Kb) of the mRNA. *P*-value was calculated using Wilcoxon rank sum test. **b** CDS SNP density in cancer-related genes and other genes. The density is calculated by the number of CDS SNPs divided by the length (Kb) of the CDS. P-value was calculated using Wilcoxon rank sum test. **c** Heatmaps displaying the number of exonic SNPs in each functional category. The number (n) is transferred with log_2_(n + 1) for the pulchritude of the graph. “onco” denotes cancer-related genes; “non-onco” denotes other genes. **Figure S2.** Dilemma of cancer-related genes. **a** Correlation between delta codon bias and nsy/syn ratio of all genes. Genes were divided into ten bins with increasing nsy/syn ratio. **b** Correlation between delta codon frequency and nsy/syn ratio of all genes. Genes were divided into ten bins with increasing nsy/syn ratio. **c** The top 20 cancer-related genes with the highest delta codon bias and relatively low nsy/syn ratio. These genes were required to have at least two synonymous and at most two nonsynonymous SNPs. **d** Diagram displaying the locations of nonsynonymous and synonymous SNPs on CDSs of the 20 genes mentioned above. Nonsynonymous and synonymous SNPs were labeled by red and blue asterisks, respectively. “onco” denotes cancer-related genes; “non-onco” denotes other genes. **Figure S3.** Detecting the constraint in cancer-related genes using highly expressed genes. **a** Comparison between the nonsynonymous to synonymous ratio (nsy/syn) of cancer-related genes and other genes. *P*-value was calculated using Wilcoxon rank sum test. **b** Comparison between the pooled nsy/syn ratio of cancer-related genes and other genes. P-value was calculated using Fisher’s exact test. **c** Conservation level (phyloP score) of nonsynonymous and synonymous SNPs in cancer-related genes and other genes. *P*-values were calculated using Wilcoxon rank sum tests. **d** Correlation between the delta codon bias after the synonymous change and the fraction of cancer-related genes. Genes were divided into ten bins with increasing delta codon bias. “onco” denotes cancer-related genes; “non-onco” denotes other genes. **Figure S4.** Detecting the constraint in cancer-related genes using genes with high GC content. **a** Comparison between the nonsynonymous to synonymous ratio (nsy/syn) of cancer-related genes and other genes. P-value was calculated using Wilcoxon rank sum test. **b** Comparison between the pooled nsy/syn ratio of cancer-related genes and other genes. P-value was calculated using Fisher’s exact test. **c** Conservation level (phyloP score) of nonsynonymous and synonymous SNPs in cancer-related genes and other genes. P-values were calculated using Wilcoxon rank sum tests. **d** Correlation between the delta codon bias after the synonymous change and the fraction of cancer-related genes. Genes were divided into ten bins with increasing delta codon bias. “onco” denotes cancer-related genes; “non-onco” denotes other genes. **Figure S5.** Detecting the constraint in cancer-related genes using genes with low GC content. **a** Comparison between the nonsynonymous to synonymous ratio (nsy/syn) of cancer-related genes and other genes. *P*-value was calculated using Wilcoxon rank sum test. **b** Comparison between the pooled nsy/syn ratio of cancer-related genes and other genes. *P*-value was calculated using Fisher’s exact test. **c** Conservation level (phyloP score) of nonsynonymous and synonymous SNPs in cancer-related genes and other genes. P-values were calculated using Wilcoxon rank sum tests. **d** Correlation between the delta codon bias after the synonymous change and the fraction of cancer-related genes. Genes were divided into ten bins with increasing delta codon bias. “onco” denotes cancer-related genes; “non-onco” denotes other genes. (PDF 700 kb)

## Abbreviations

CDS: Coding sequence
CUB: Codon usage bias
NGS: Next generation sequencing
SNP: Single nucleotide polymorphism
UTR: Untranslated region
