# Supplementary material for: Nonsynonymous, synonymous and nonsense mutations in human cancer-related genes undergo stronger purifying selections than expectation
Source: BMC Cancer. 2019 Apr 16;19:359. doi: 10.1186/s12885-019-5572-x (PMC6469204; doi:10.1186/s12885-019-5572-x)

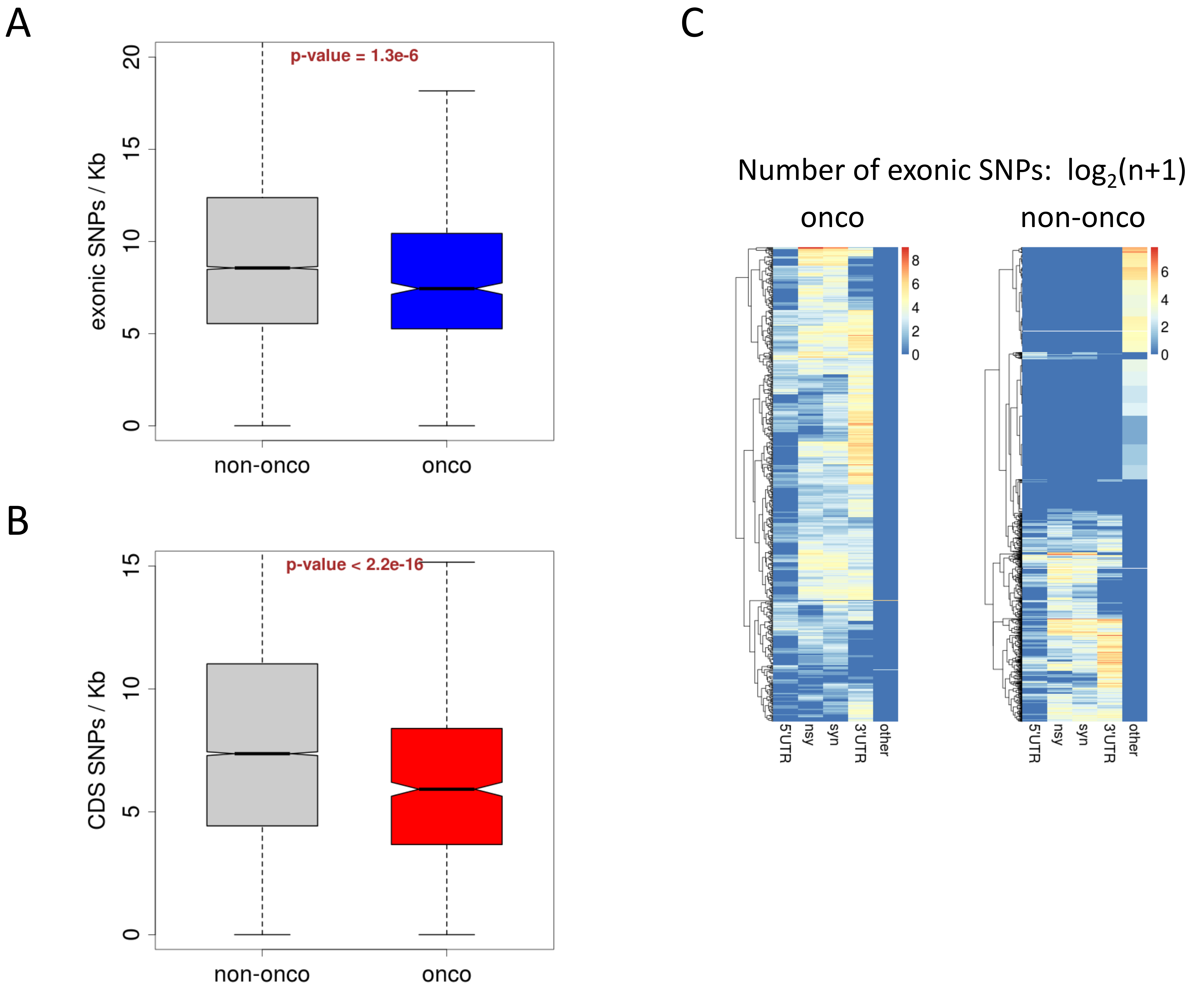

A
B
C
p-value = 1.3e-6
exonic SNPs / Kb
0
5
10
15
20
non-onco
onco
p-value < 2.2e-16
CDS SNPs / Kb
0
5
10
15
non-onco
onco
Number of exonic SNPs: log2(n+1)
onco
non-onco
8
6
4
2
0
6
4
2
0
5'UTR
nsy
syn
3'UTR
other
5'UTR
nsy
syn
3'UTR
other

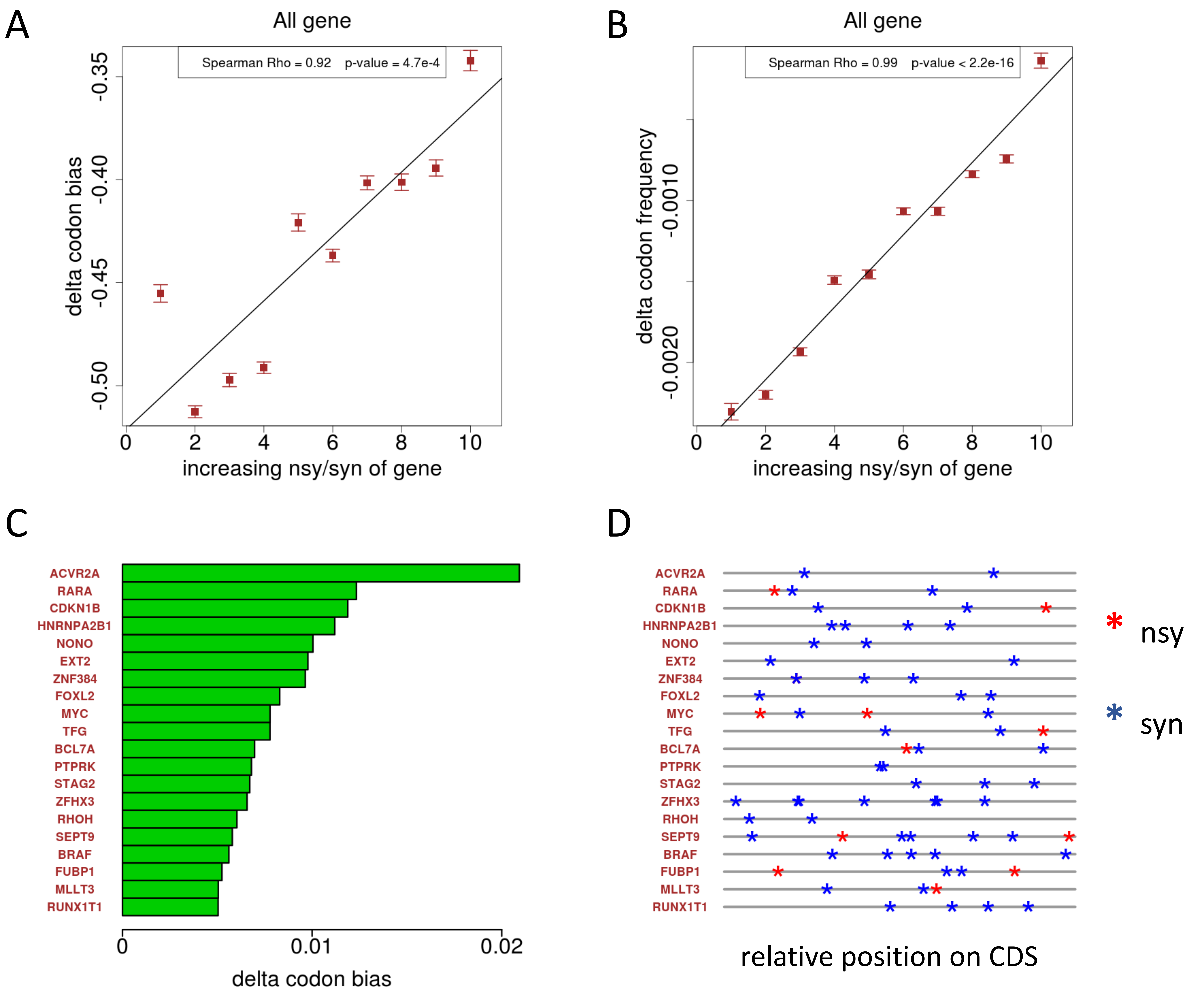

A
All gene
Spearman Rho = 0.92 p-value = 4.7e-4
delta codon bias
-0.35
-0.40
-0.45
-0.50
0
2
4
6
8
10
increasing nsy/syn of gene
B
All gene
Spearman Rho = 0.99 p-value < 2.2e-16
delta codon frequency
-0.0010
-0.0020
increasing nsy/syn of gene
C
ACVR2A
RARA
CDKN1B
HNRNPA2B1
NONO
EXT2
ZNF384
FOXL2
MYC
TFG
BCL7A
PTPRK
STAG2
ZFHX3
RHOH
SEPT9
BRAF
FUBP1
MLLT3
RUNX1T1
0
0.01
0.02
delta codon bias
D
nsy
syn
relative position on CDS

Highly expressed genes

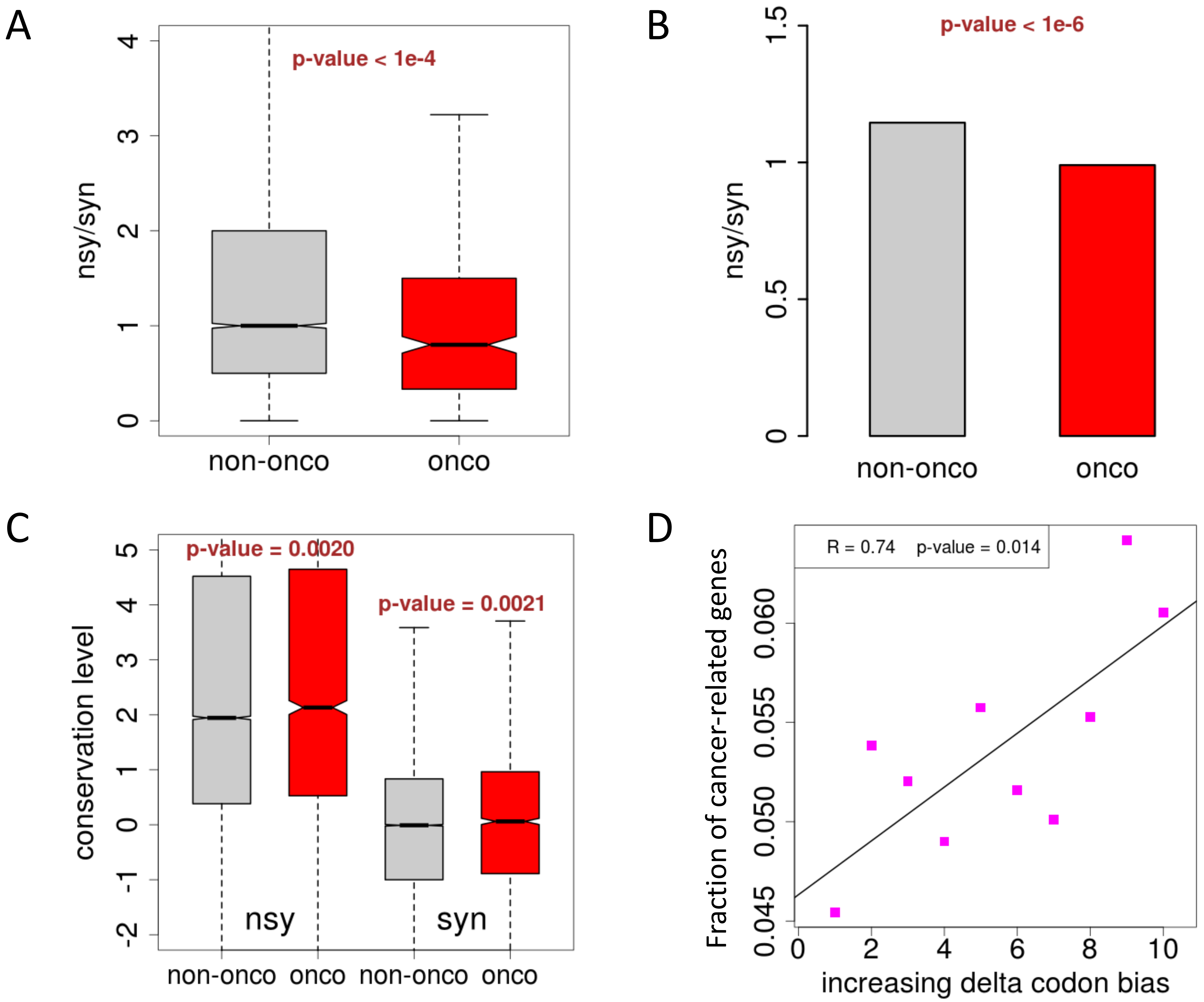

Genes with high GC content

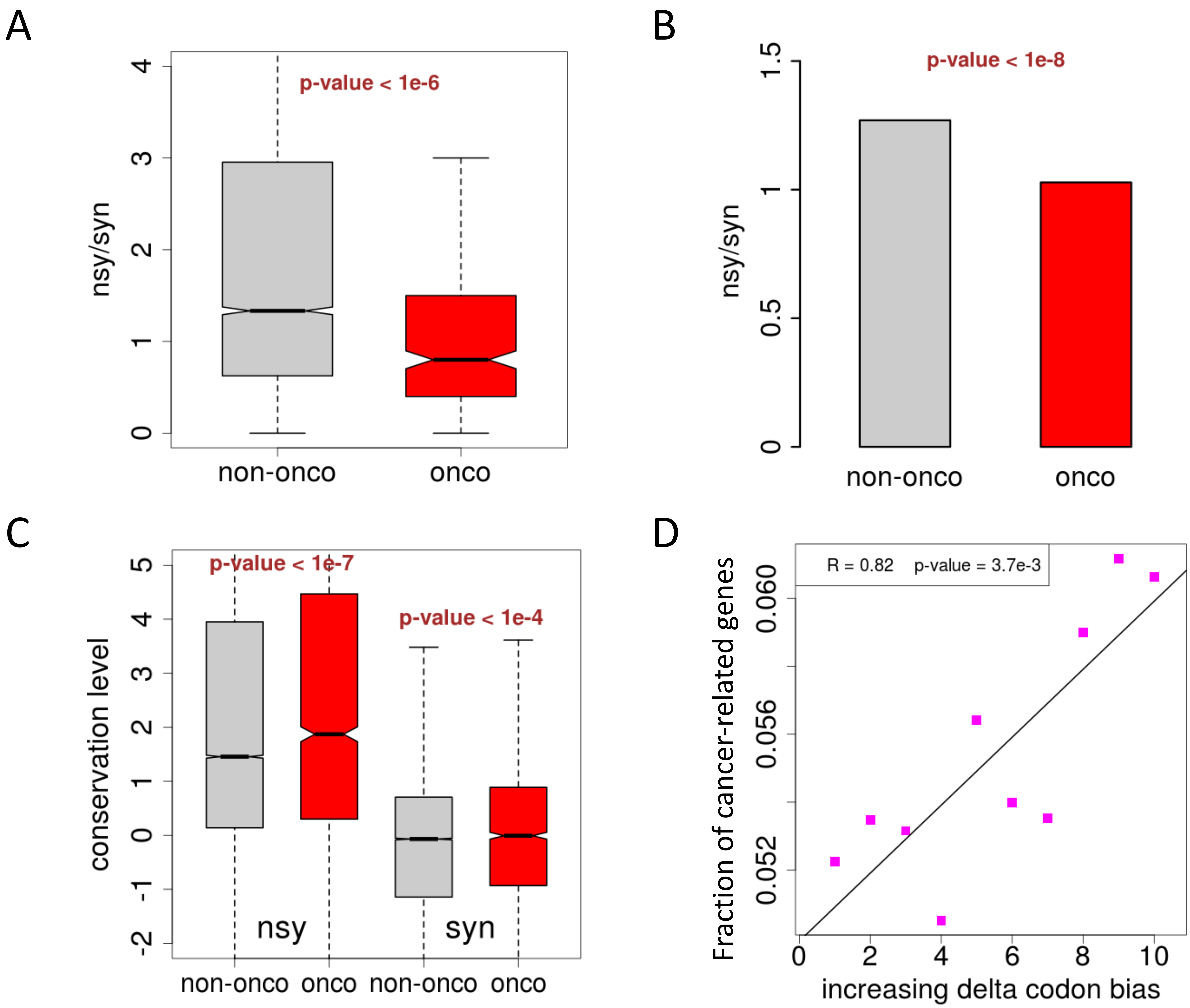

Genes with low GC content

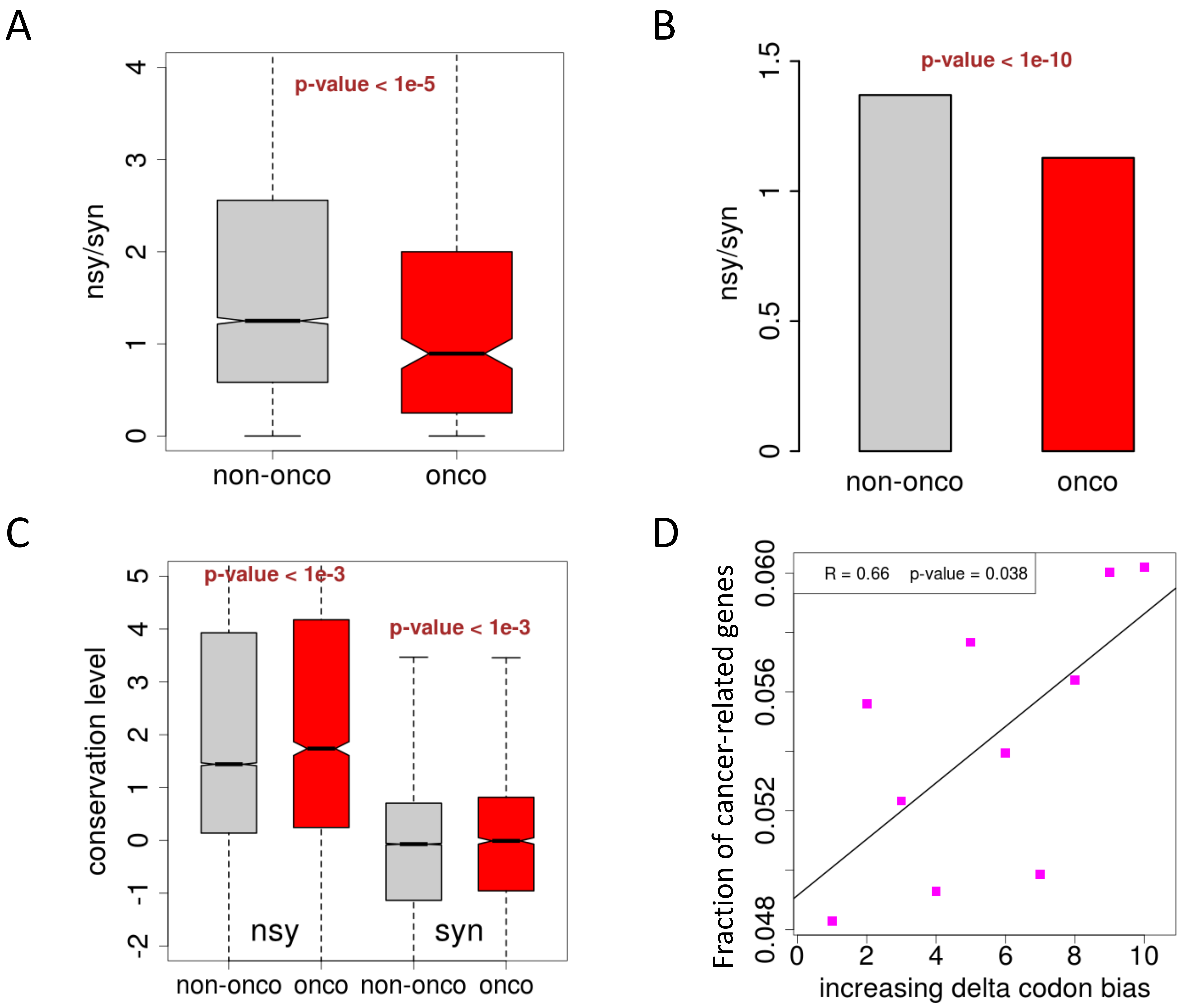

Supplement: Supplementary file 3 — Figure S1. Profiling the SNPs in cancer-related genes and other genes. a Exonic SNP density in cancer-related genes and other genes. The density is calculated by the number of exonic SNPs divided by the length (Kb) of the mRNA. P-value was calculated using Wilcoxon rank sum test. b CDS SNP density in cancer-related genes and other genes. The density is calculated by the number of CDS SNPs divided by the length (Kb) of the CDS. P-value was calculated using Wilcoxon rank sum test. c Heatmaps displaying the number of exonic SNPs in each functional category. The number (n) is transferred with log2(n + 1) for the pulchritude of the graph. “onco” denotes cancer-related genes; “non-onco” denotes other genes. Figure S2. Dilemma of cancer-related genes. a Correlation between delta codon bias and nsy/syn ratio of all genes. Genes were divided into ten bins with increasing nsy/syn ratio. b Correlation between delta codon frequency and nsy/syn ratio of all genes. Genes were divided into ten bins with increasing nsy/syn ratio. c The top 20 cancer-related genes with the highest delta codon bias and relatively low nsy/syn ratio. These genes were required to have at least two synonymous and at most two nonsynonymous SNPs. d Diagram displaying the locations of nonsynonymous and synonymous SNPs on CDSs of the 20 genes mentioned above. Nonsynonymous and synonymous SNPs were labeled by red and blue asterisks, respectively. “onco” denotes cancer-related genes; “non-onco” denotes other genes. Figure S3. Detecting the constraint in cancer-related genes using highly expressed genes. a Comparison between the nonsynonymous to synonymous ratio (nsy/syn) of cancer-related genes and other genes. P-value was calculated using Wilcoxon rank sum test. b Comparison between the pooled nsy/syn ratio of cancer-related genes and other genes. P-value was calculated using Fisher’s exact test. c Conservation level (phyloP score) of nonsynonymous and synonymous SNPs in cancer-related genes and other genes. [file 12885_2019_5572_MOESM3_ESM.pdf]
